# Medical Attendance upon Servants

**Published:** 1898-05-07

**Authors:** 


### MEDICAL ATTENDANCE UPON SERVANTS.

In his summing up in the case of Mrs. Nicholls, charged with the manslaughter of Emily Jane Popejoy, the judge, Mr. Justice Phillimore, made some remarks about the provision of medical attendance for servants which are of considerable interest to medical practitioners. It is generally understood that a medical man attending a servant has no claim upon her mistress unless the attendance is given at her request. This often gives rise to great injustice. A medical man receives a message, which, as he has every reason to believe, comes from the employer, asking him to visit a servant. In response to this message he attends, and on applying for his fees he is referred to the servant, who perhaps has by that time left her place, and, in any case, cannot be followed up. He may even see the mistress on each occasion, but he has no claim unless he can show that he has been asked by the mistress to attend, which, of course, he cannot do. On the other hand, it is a distinct hardship upon a servant that for every trivial ailment she should be expected to call in the doctor that attends the family, often a man of some standing whose fees are fairly high, when she could get all she wants by going to the nearest red lamp round the corner. In any case, the position is a difficult one. It is rendered still more complicated, however, by the definite ruling of the judge, in the case referred to, that, although it is not the duty of the employer to pay for medical attendance upon a servant, it is his or her duty to send for a doctor or to procure medical aid. The suggestion is made that the poor-law medical officer is always available, but we much doubt whether the guardians will see the matter in the same light. The following are the words used by the judge, as reported in the *Times:* "There was a duty to send for a doctor or to procure medical aid. He did not wish to carry that statement too far, as it was an important question of law. It was not the duty of a master or mistress to pay for a doctor for a servant. It was in the case of an apprentice, not a servant; but it was the duty of the parish to provide a doctor for a servant who had not the means. It was not the duty of a mistress to provide a doctor in the sense of hiring a doctor and paying for him at her own expense, but it was her duty if she saw that the girl was seriously ill—if she knew, or ought to have known, that the girl was seriously ill—to send for some one, because it was her duty to see that the girl had medical aid, which the law of the country provided for every one. In his opinion, if the jury thought the circumstances were such that a doctor ought to have been called in, and the prisoner knew these circumstances, then there was a duty on the part of the prisoner to put the parish doctor in communication with the girl, or the girl in communication with the parish doctor." Just so. But will the parish doctor go? and will the parish undertake this attendance upon servants? It is the duty of the mistress to "send for a doctor, or to procure medical aid," but it is not her duty to pay for it. Who, then, is to pay? It is clearly quite impossible for a mistress to know that her servant is "without means" in the sense that would entitle her to parish aid, and she clearly cannot send for any other medical aid without rendering herself liable, which the judge definitely says she is not bound to do.

